# Suicidal Thoughts and Behaviors Among Health Care Trainees, Staff and Faculty at an Academic Medical Center

**DOI:** 10.3390/jcm14020574

**Published:** 2025-01-17

**Authors:** Sijia Zhang, Sidney Zisook, Judy Davidson, Desiree Shapiro, Neal Doran

**Affiliations:** 1 School of Medicine, University of California, San Diego, CA 92093, USA; 2 Department of Psychiatry, University of California, San Diego, CA 92093, USA; szisook@health.ucsd.edu (S.Z.); dlshapiro@health.ucsd.edu (D.S.); nmdoran@health.ucsd.edu (N.D.); 3 Health Sciences, University of California, San Diego, CA 92121, USA; jdavidson@health.ucsd.edu; 4 Mental Health Care Line, VA San Diego Healthcare System, San Diego, CA 92161, USA

**Keywords:** suicide, suicide risk, depression, intense affective states, health care workforce, mental health care, workplace wellness

## Abstract

**Background/Objectives**: Health care workers are at greater risk for death by suicide compared to the general population and are less likely to seek assistance. More information about correlates of suicidality and treatment-seeking behavior are needed to reduce risk. **Methods**: The American Foundation for Suicide Prevention developed an Interactive Screening Program to identify and engage at-risk staff and trainees in health care settings. The study reports on the prevalence and demographic and clinical predictors of current suicidal thoughts, behaviors and mental health treatment at a single site (*n* = 5898) from 2009 to 2024. **Results**: The study found that 18.2% of respondents reported current suicidal thoughts and behaviors. These were more common among respondents who were younger, male, and who identified as a race/ethnicity other than non-Hispanic White. Suicidal thoughts and behaviors were more likely among those with higher PHQ-8 scores (*OR* = 1.23, *p* < 0.01) and those who endorsed maladaptive coping behaviors, hopelessness, loneliness, stress and nervousness (*OR*s 1.36–3.04, *p*s < 0.01). Current mental health treatment was more likely among women, non-Hispanic White respondents compared with Asian or Pacific Islander respondents, and nurses relative to physicians. Mental health treatment was also associated with higher PHQ-8 scores, lifetime suicide attempts, difficulty controlling eating and alcohol consumption, and recent feelings of anxiety, stress and nervousness. **Conclusions**: Findings suggest a continued need to identify and engage health care trainees and staff who are at risk for suicide and to establish new approaches to linking these individuals to resources or interventions aimed at reducing risk. The study identified male and/or Asian/Pacific Islander-identifying health care workers who reported intense loneliness and/or hopelessness, use of non-prescription drugs and recent suicidal thoughts and/or behaviors as high-risk individuals who may require enhanced methods of outreach, identification, acceptance and accessibility of treatment.

## 1. Introduction

Most people who experience suicidal ideation (SI) do not die by suicide and many individuals who die by suicide have never reported SI [1]. However, failure to identify SI may be a missed opportunity for prevention, especially when SI occurs in the context of other risk factors. One known risk for suicide is occupation [2], and the health care workforce is an “at-risk” group [3,4,5]. Substance use, intense affective states, untreated depression and suicidal thoughts and behaviors are highly prevalent among those who learn and work in health care settings, including students, physicians, nurses and other members of the health care workforce. Each of these conditions, in turn, increases the risk for suicide [6]. However, most health care trainees and workers struggling with mental health concerns do not seek the support they need [7,8].

The American Foundation for Suicide Prevention’s (AFSP) Interactive Screening Program (ISP) was developed to screen medical students, trainees, health staff and physicians for distress and suicide risk, to engage those at risk and to offer a confidential way for them to connect with mental health services [9,10]. Individuals are invited to complete a brief, web-based, anonymous stress and depression questionnaire, after which they receive a personalized response from a mental-health-trained counselor. An evaluation of six medical schools using the ISP reported that 98% of 1413 participants (medical students, residents and faculty physicians) were designated as having high or moderate distress, yet only 5% were receiving counseling or therapy [9]. A report on nurses using the ISP found similarly alarming rates of high distress and insufficient mental health treatment [11]. Thus, there remains an urgent need to identify health care workers at risk and connect them with appropriate resources.

There are limited existing studies identifying the risk factors for suicidal thoughts and behaviors among health care trainees and staff. This report describes suicidal thoughts and behaviors and mental health treatment of health care students, trainees, staff and faculty who participated in the UC San Diego screening program from 1 May 2009 through 14 June 2024. Previous reports from this institution have documented high rates of depression, burnout and suicidal ideation among medical students, house staff, faculty and nurses, and low rates of mental health care even among those at highest risk [12,13,14,15]. Over time, the program has dramatically increased participation, expanded services to additional health care workers and has weathered the COVID-19 storm. In this report, we explore differences in the frequency of suicidal thoughts and behaviors and the utilization of mental health resources among various sub-groups in our health care trainee and worker population who completed the screening questionnaire. We provide a novel examination of a broad range of predictors for suicidal thoughts and behaviors among health care trainees and workers, including demographics, professional role, intense affective states and maladaptive coping behaviors. Our ultimate goal is to identify actionable targets for suicide prevention among the vulnerable health care community.

## 2. Materials and Methods

### 2.1. Overview

The AFSP’s ISP has been the key component of UC San Diego’s health care workforce suicide prevention program since 2009. The program’s initial target audience included all faculty physicians, residents and medical students. In the second year of the program, 2010, it expanded to include pharmacy students. In 2016, there was a broader expansion that included nursing staff and the entire health system staff, from health professionals to support personnel. Email invitations describing the program were sent at least annually to health students, trainees, staff and faculty. The invitations assured recipients of its confidentiality, encouraged individuals to participate in the anonymous web-based screen and provided links to the Healer Education Assessment and Referral (HEAR) program website and the screening tool [10]. A Master’s-level program counselor reviewed all questionnaire responses and directly responded to the participants with a customized response based on the level of distress, depression and/or suicide risk indicated by the participants’ survey responses [10,16]. The UC San Diego Institutional Review Board (IRB) determined the project to be Not Human Subject Research and excluded the study from IRB review and waived the need for participant informed consent.

### 2.2. Participants

UC San Diego Health-affiliated physicians, nurses, trainees, students and other staff (e.g., pharmacists, social workers, allied health, hospital staff) who completed the online Stress and Depression Questionnaire between 1 May 2009 and 14 June 2024 (*n* = 5898) were included in our program evaluation. It is important to note that the HEAR Program was primarily developed as an outreach and education effort to destigmatize help-seeking and prevent suicide, and therefore those who chose to participate did so not for the purpose of research participation, but out of personal interest for services that the program provided.

### 2.3. Instrument

The HEAR Stress and Depression questionnaire contains the 9-item Patient Health Questionnaire (PHQ-9) [17]; measures of intense affective states (worrying, irritability, anxiety, loneliness, anger, etc.) that have been linked to depression with suicidal ideation; alcohol and drug use; disordered eating behaviors; current suicidal thoughts, behaviors, and past suicide attempts; self-harm; current psychiatric treatment; and age, gender, race or ethnicity. In order to optimize the anonymity of the questionnaire for respondents who were concerned, demographic questions were optional. A final optional item asked participants to provide an email address, which would be encrypted to facilitate anonymous communication with the program counselors through the website. All resulting data used for analysis were de-identified.

#### 2.3.1. Suicidal Thoughts and Behaviors

Participants were asked questions including “during the last two weeks, how often have you...had thoughts about taking your own life? Planned ways of taking your life? Done things to hurt yourself?” Participants were asked to select a response from a four-point scale (0—not at all, 1—some of the time, 2—a lot of the time, 3—most or all the time). For our purposes, respondents were considered as currently having suicidal thoughts and behaviors if they gave a rating of 1 or higher (i.e., at least some of the time) on any of these three items, or on item 9 of the PHQ-9 (“having thoughts that you would be better off dead or thoughts of physically harming yourself” in the last two weeks) [18]. Additionally, respondents were asked if they had ever made a lifetime suicide attempt.

#### 2.3.2. Depression Symptoms and Severity

Participants completed the nine-item Patient Health Questionnaire depression scale (PHQ-9) [17,19]. The PHQ-9 items mirror the Diagnostic and Statistical Manual, 5th edition (DSM-5) criteria for major depression [20]. Participants were asked to select a response from a 4-point scale (0—not at all, 1—some of the time, 2—a lot of the time, 3—most or all the time) that best describes how they felt during the past two weeks [17]. The severity of depression symptoms was operationalized as the sum of scores on the first eight items (PHQ-8); the ninth item evaluating thoughts of self-harm was excluded here because it was incorporated into the suicidal behaviors outcome as described above. The PHQ-8 has been found to be a valid and reliable measure of depression severity and has been found to be comparable to the PHQ-9 as a diagnostic measure for DSM-IV major depression [17]. Data from a representative sample of nearly 200,000 individuals in the United States support the value of the PHQ-8 in population studies [21].

#### 2.3.3. Associated Intense Affective States

Participants were asked to rate on a four-point scale (0—not at all, 1—some of the time, 2—a lot of the time, 3—most or all the time) how often they experienced the following intense affective states over the past four weeks: “feeling nervous or worrying a lot; becoming easily annoyed or irritable; feeling your life is too stressful; having arguments or fights; feeling intensely anxious or having anxiety attacks; feeling intensely lonely; feeling intensely angry; feeling hopeless; feeling desperate; feeling out of control”. Each of these affective states has been found to signal suicidal crisis in depressed patients [22]. Responses were transformed into binary variables reflecting low (“not at all” or “some of the time”) versus high (“a lot of the time” or “most or all of the time”) levels of each affective state.

#### 2.3.4. Alcohol, Substance Use and Disordered Eating Behaviors

Participants were asked if they had engaged in or experienced the following over the past four weeks: “drinking alcohol (including beer or wine) more than usual; using drugs other than alcohol (marijuana, cocaine, etc.); feeling that you can’t control what or how much you eat”. Response options were given on a four-point scale (0—not at all, 1—some of the time, 2—a lot of the time, 3—most or all of the time). Responses were transformed into binary variables reflecting low (“not at all” or “some of the time”) versus high (“a lot of the time” or “most or all of the time”) levels of each behavior.

#### 2.3.5. Current Mental Health Treatment

Participants were asked if they were currently taking prescribed medications for anxiety, depression or stress, and if they were receiving counseling or therapy. Responses were combined into a single binary item reflecting whether participants were receiving any mental health treatment or no treatment.

### 2.4. Procedure

Individuals who participated in the interactive screening program (ISP) created an anonymous online account to complete the survey. Following procedures previously described [16,23], once participants completed and submitted their surveys, a computer program automatically generated a PHQ-9 score and used this, along with responses to other items, to classify respondents into one of three tiers. Criteria for Tier 1 (high risk) included current suicidal thoughts and behaviors; a PHQ-9 score of 15 or higher with feelings of intense anxiety, anger, hopelessness, desperation, or feeling out of control “a lot of the time” or “most of the time” or feelings of nervousness, annoyance, stress, loneliness, or having arguments “most of the time”; and a PHQ-9 score of 10–14 with a history of prior suicide attempts, active problems related to alcohol or drug use, disordered eating behaviors or indications that current problems were making it somewhat difficult to function. Criteria for Tier 2 (moderate risk) included a PHQ-9 score of 10–14 without a history of suicide attempts or current suicidal thoughts and behaviors, problems related to alcohol or drug use or eating behavior or an indication that current problems were making it somewhat difficult to function. Respondents who did not meet any of these criteria were designated as Tier 3 (low risk). Tier classification determined the recommendations for further follow-up, evaluation and support provided to participants. Tier 1 designation is included in Table 1 for descriptive purposes, but the tier variable was not included in analyses in the current manuscript.

### 2.5. Statistical Approach

Descriptive statistics were used to characterize the study sample. Separate binary logistic regression models were used to test the associations between four families of predictors (demographics, high-risk indicators, maladaptive coping behaviors and intense affective states) and two outcomes (current suicidal thoughts and behaviors, currently receiving mental health treatment). To reduce the potential for confounding, demographic predictors were included as covariates in models testing other families of predictors. Stata 17.0 (StataCorp LLP, College Station, TX, USA) was used for all analyses. Given the number of significance tests planned a priori*,* a family-wise alpha correction was used to adjust the alpha level for statistical significance to *p* < 0.0125.

## 3. Results

A total of 5898 participants completed the ISP questionnaire between 1 May 2009 and 14 June 2024. The mean age was 37 years, with 31.5% over the age of 40. Most respondents were female (70.7%). Approximately half identified as non-Hispanic White (50.6%), 17.9% as Asian or Pacific Islander, 10.9% as Latino, 2.5% as Black and 18.2% as “other” (includes American Indian or Alaskan native; multiracial; prefer not to answer). Almost one quarter of the participants were nurses (22.3%), while 16.8 % and 14.6% were residents and fellows, 11.5% medical students, 6.3% faculty or staff physicians, 5.26% pharmacy students and 38.8% “other” (PhD faculty; other faculty; pharmacists; other clinical and nonclinical staff, prefer not to answer; and no answer).

Assessing depression severity, the average PHQ-8 score was 8.6, which falls in the mild depression range. Over a third (38.7%) had a PHQ-8 score > 10 (at least moderately severe). The most frequently endorsed intense affective states were nervousness (51.6%), stressed (50.5%), and annoyed (42.4%). More than one quarter endorsed anxiety (26.3%) and almost that many felt intensely lonely (24.1%), while 19.8% felt hopeless and 14.6% desperate. Just over one quarter of all participants (26.8%) were receiving counseling, therapy or medication for depression, anxiety or stress at the time of completing the screening questionnaire. Detailed descriptive clinical and treatment data are shown in Table 1.

Table 2 provides data on the frequencies of current suicidal thoughts and behaviors, and designation as Tier 1 (high risk) by demographics, role in the health care system and depression severity. Of note, more than 50% of females (53.1%), Hispanics (58.0%), Asians/Pacific Islanders (55.8%), nurses (58.4%) and “other” (51.9%) were categorized as high risk. Fewer participants (but still a considerable number) endorsed current suicidal thoughts and/or behaviors, with 18.4% of those under 40 years of age, 19.5% males, 20.8% Asians/Pacific Islanders, 19.6% nurses, and 36% of those with a PHQ-8 score ≥ 10.

Table 3 provides data on the frequencies of current mental health treatment (current medications for depression, anxiety and/or stress; current mental health counseling or psychotherapy; current medications and/or therapy) by demographics, role in the health care system and depression severity. Of note, only approximately one third of participants (35.9%) with a PHQ-8 score ≥ 10 reported current mental health treatment with medication and/or therapy. While 31.1% of Non-Hispanic White-identifying health care workers reported current mental health treatment, only 18.0% of Asian or Pacific Islander-identifying health care workers reported current mental health treatment.

### 3.1. Prediction of Current Suicidal Thoughts and Behaviors

Nearly one in five participants (18.2%) endorsed at least one item reflecting current suicidal thoughts and behaviors. The full model of the association between demographic predictors and current suicidal thoughts and behaviors is shown in Supplementary Table S1. There was a significant inverse association with age [odds ratio (OR) = 0.98 (95% ci 0.97, 0.99)], such that each additional year of respondent age was associated with a 2% reduction in the likelihood of endorsing current thoughts or behaviors. Current thoughts and behaviors were also 20% less likely among respondents who identified as female compared with those who identified as male [OR = 0.80 (0.68, 0.94)]. In terms of racial/ethnic background, current thoughts and behaviors were 38% more common among those who identified as Asian or Pacific Islander [OR = 1.38 (1.14, 1.68)] and 45% more common among those who identified as being from other or multiple backgrounds [OR = 1.45 (1.15, 1.82)] relative to those who identified as non-Hispanic White. There were no significant differences between physicians and any other health care roles.

Table 4 summarizes the associations between predictors of interest and current suicidal thoughts and behaviors in the three additional models. In the depression model, both predictors (PHQ-8 score and history of lifetime suicide attempts) were significantly associated with current thoughts and behaviors. Each additional point on the PHQ-8 scale predicted a 23% increase in the likelihood of current suicidal thoughts and/or behaviors. Similarly, those who reported having made a lifetime suicide attempt were 217% more likely to also endorse current thoughts and behaviors. In the model of the association between maladaptive coping behaviors and current suicidal thoughts and behaviors, all hypothesized predictors were significantly associated with suicidal thoughts and behaviors. More specifically, the endorsement of drinking more than usual, using non-prescription drugs other than alcohol and having difficulty controlling eating behaviors was associated with 107%, 161% and 121% greater odds of current suicidal thoughts and behaviors, respectively.

The model of the association between intense affect and current suicidality indicated that some affective states were stronger predictors of suicidality than others. Hopelessness and loneliness exhibited the strongest associations with suicidal thoughts and behaviors. Individuals who endorsed these states were 204% and 148%, respectively, more likely to also endorse current suicidality, compared with those who did not endorse those states. Additionally, the endorsement of stress and nervousness predicted 87% and 36% greater likelihood of current suicidality. In contrast, annoyance, having arguments or fights, anxiety, desperation and feeling out of control were not significantly associated with current suicidality.

### 3.2. Predictors of Mental Health Treatment

The associations between demographic predictors and the odds of receiving mental health treatment are shown in Supplemental Table S2. Analyses indicated that women were 42% more likely than men to be in treatment [OR = 1.42 (1.22, 1.66)]. Respondents who identified as Asian or Pacific Islander were 47% less likely to be in treatment compared to those who identified as non-Hispanic White [OR = 0.53 (0.44, 0.64)]; no other racial or ethnic groups differed significantly from the non-Hispanic White group. In terms of position, nurses were 58% more likely than physicians to be receiving mental health care [OR = 1.58 (1.18, 2.14)]. No other position groups were significantly different from physicians. Age was also not significantly associated with treatment status.

Table 5 summarizes the three additional models predicting the likelihood of mental health treatment. Each model included age, gender, racial/ethnic background, and role as covariates. In the depression and risk model, each additional point on the PHQ-8 was associated with 8% greater odds of receiving mental health treatment. Respondents who reported lifetime suicide attempts were 76% more likely to be in treatment compared with those who did not. Current suicidal thoughts and behaviors were not significantly associated with treatment status. In the maladaptive coping model, difficulty controlling eating behaviors was associated with an 86% greater likelihood and drinking more than usual with a 50% greater likelihood of receiving mental health care. Other substance use was unrelated to treatment status.

In the model of intense affective states, those who endorsed anxiety were 59% more likely to be receiving mental health treatment. Respondents who endorsed stress had 30% greater odds of being in treatment compared with those who did not. Similarly, endorsement of nervousness was associated with 25% greater odds of being in treatment. Endorsement of other affective states was not associated with the likelihood of being in mental health treatment.

## 4. Discussion

In this report, we aimed to provide a comprehensive evaluation of suicidal thoughts and behaviors among health care students, trainees, and workers over a 15-year period. Our findings highlight not only the alarming presence of suicidal thoughts and behaviors among health care workers and trainees, but also the concerningly low rates of mental health treatment-seeking within this population. Through examining the associations between suicidal thoughts and behaviors and demographic information, health care role, depression severity, intense affective states and maladaptive coping behaviors, we have re-confirmed known risk factors and identified new predictors to identify health care workers at risk for suicide. We found that health care workers as a group had low rates of mental health treatment, with certain subgroups, including those identifying as Asian or Pacific Islander, males and physicians, even less likely to seek mental health treatment. Overall, the pattern of findings suggests that health care workers’ ability and/or willingness to access mental health care may be substantially limited by sociocultural and demographic factors even when a clinical need exists. These results have critical implications for the ongoing need to address the mental health crisis in health care workers and trainees, especially among those with identifiable risk factors for current suicidal thoughts and behaviors.

We found that almost one fifth of the health care workforce (18.2%) who participated in UC San Diego’s ISP screening were struggling with current suicidal thoughts and behaviors. It is well established that health care professionals are disproportionately faced with mental health challenges [24,25], which are influenced by grueling occupational hours and exposure to traumatic workplace events. Echoing previous works in the literature, suicidal thoughts and behaviors were associated with higher depression severity, a history of lifetime suicide attempts, male gender and maladaptive coping behaviors such as misuse of alcohol and drugs and disordered eating behaviors [26,27,28]. In a cohort study evaluating data from the National Violent Death Reporting System in the United States, A review of data from the National Violent Death Reporting System found that health care professionals who died by suicide were more likely to have Asian or Pacific Islander ancestry compared with individuals in the general population who died by suicide [29]. Consistent with this, we found that Asian or Pacific Islander health care workers were more likely to be experiencing current suicidal thoughts or behaviors than their non-Hispanic White counterparts, but less likely to be receiving mental health care. We identified several intense affective states to be associated with current suicidal thoughts and behaviors: hopelessness, loneliness, stress and nervousness. While hopelessness and loneliness have previously been established to be linked to a suicidal crisis [12,30], we are unaware of prior studies identifying the intense affective states of stress and nervousness as additional risk factors. Recognizing intense affective states among health care workers may help direct attention and support to those at risk for experiencing current suicidal thoughts and behaviors. Online screening tools and peer reports are potential interventions to identify health care trainees and workers with concerning intense affective states or other predictors of suicidal thoughts and behaviors [31]. The anonymous encryption of the ISP overcomes issues associated with psychological safety and may enhance acceptability and access to those who might not have otherwise sought treatment [23,32].

Although over half the participants rated themselves as nervous (51.6%) and/or stressed (50.5%), depression severity scores were high and most participants were classified as high risk (51.1%), only about a quarter (26.8%) were receiving any mental health treatment. Our finding that the majority of health care students and providers were not receiving mental health care was no surprise. It is well known that despite their knowledge and resources, health care workers are reluctant to seek mental health care [32]. One of the most well-established and effective methods to prevent suicide is the treatment of depression [33], yet the majority of physicians and nurses with depression do not seek professional care [15,34] and large United States studies have found that only a minority of individuals who die by suicide had recently been in psychiatric care [35,36,37]. One reason for under-treatment among health care professionals has been attributed to intrusive questions regarding mental health treatment and history used by licensure and accreditation boards. These stigmatizing questions have resulted in many avoiding treatment for mental health conditions, sometimes leading to self-medication through substance use [38,39].

In the current study, it was somewhat reassuring to find past suicide attempts and high depression symptom severity to be associated with receiving mental health treatment, as well as endorsements of high levels of stress, nervousness and anxiety. However, it was concerning that current suicidal thoughts and behaviors and feelings of helplessness and desperation were not. To our knowledge, these important correlates of mental health treatment in health care workers have not been previously reported. This study is also consistent with previous studies in the general population [40], in that females were more likely to receive treatment than males, which may at least partially explain why nurses—predominantly (86.6%) female—were more likely than physicians to receive treatment. We are not aware of previous studies reporting that Asian/Pacific Islander health care workers were less likely than White health care workers to receive mental health care, although this mimics health care disparities observed in community populations [41,42]. More research is needed to understand the specific barriers for mental health service usage among minoritized groups in the health care worker population, but possibilities include cultural and community contributions to mental health stigma and mismatch between cultural needs and available services [43]. Potential interventions to address cultural mental health disparities include adapting assessment procedures to reflect cultural variations in symptom expression, employing language-proficient and cultural humility-trained providers and decreasing stigma and professional consequences for seeking treatment.

This study has several important limitations. As the sample was limited to one university-based, academic medical center in southern California, demographic representation may not reflect the broader national health care workforce. Not all health care students, trainees and professionals took this screening. Participants opted to complete the depression and stress screening, which may have introduced a selection bias by attracting individuals who were distinct from peers and had pre-existing concerns for their mental health. Additionally, the ISP questionnaire is not designed to be a research tool but rather a screening instrument to identify individuals who would benefit from referral to mental health treatment. A wider and perhaps more representative and impactful utilization of this screening and engagement tool might be to include it in initial orientation and onboarding procedures. Underrepresented groups such as Black/African American or gender non-binary individuals had small sample sizes, which may have masked significant associations. Program participants also may have chosen not to complete demographic items in order to protect their identity, and therefore findings based on demographic data need to be further confirmed with prospective studies. Future studies could also employ broader sampling strategies by distributing the screening tool to health care trainees and workers across multiple institutions. Our analysis of current suicidal thoughts and behaviors did account for the intent, severity or immediacy of suicidal thoughts and behaviors, which would help better identify and target the most at-risk population for death by suicide.

This study also has several strengths. It uses a large sample of health care learners and professionals and takes advantage of the AFSP’s Interactive Screening Program (ISP), an evidence-based, innovative program that has been widely adopted across more than 200 institutions of higher education, medical and professional degree schools, organizations and workplaces, and has successfully connected over 280,000 individuals to professional help [44]. Our results helped identify actionable targets for suicide prevention among the health care community.

Comparing features associated with suicide risk to those associated with mental health care is one way to identify unmet needs. To that end, identifying as male and as Asian/Pacific Islander were independently associated with increased risk for suicidal thoughts and/or behaviors. However, Asians/Pacific Islanders were less likely than non-Hispanic Whites, and males less likely than females, to receive mental health treatment. Individuals with high levels of intense affective states of nervousness, stress, loneliness and hopelessness were at increased risk for suicidal thoughts and behaviors, but loneliness and hopelessness were not predicters of current treatment. A qualitative study of medical residents’ descriptions of stressors in the workplace triangulates the psychological impact of the loneliness that residents face when relocating for training. This finding suggests a need for structured socialization activities to reduce risk. Similarly, taking non-prescribed drugs was associated with suicidal thoughts and/or behaviors, but not with treatment. And perhaps most surprising, current thoughts and/or behaviors were not related to treatment. These features—being a health care worker who identifies as male and/or Asian/Pacific Islander, experiences intense loneliness and/or hopelessness, takes non-prescription drugs and endorses recent suicidal thought and behaviors—may help identify high-risk individuals who require enhanced methods of outreach, identification, acceptance and accessibility of treatment.

## 5. Conclusions

This program documented high rates of suicidal thoughts and behaviors, validated known risk factors, uncovered new risk factors and revealed suboptimal rates of mental health care utilization in medical students, physician trainees, attending physicians, nurses and others in the health care workforce. In all, almost 6000 unique individuals participated in this screening program’s online survey. We now know that the adage “physician heal thyself” can and should be extended to the entire health care family. Future research is indicated to test strategies for earlier engagement in mental health treatment for vulnerable populations within the health care workforce. The implementation of interventions such as systematized, anonymous screenings and streamlining processes to receive mental health support may encourage health care trainees and staff to prioritize and care for their mental health. Only then will health care learners and providers be able to experience the joy the practice of health care is meant to provide, and to deliver the highest level of care to their patients.

## Figures and Tables

**Table 1:** Descriptive data for the HEAR Interactive Screening Program Stress and Depression Questionnaire, 1 May 2009 to 14 June 2024 (*n* = 5898).

	*N*	%
Depression severity		
PHQ-8 ≥ 10	2284	38.72
Maladaptive coping behaviors (occurring a lot or most or all the time over the past four weeks)		
drinking alcohol more than usual	399	6.77
using non-prescription drugs other than alcohol	74	1.25
unable to control eating	1018	17.26
Intense affective states (occurring a lot or most or all the time over the past four weeks)		
nervous	3045	51.63
annoyed	2500	42.39
stressed	2977	50.48
fighting	854	14.48
anxious	1552	26.32
lonely	1419	24.06
angry	772	13.09
hopeless	1165	19.75
desperate	860	14.58
out of control	1082	18.34
Suicidality		
wish to be dead (PHQ-9 item 9 scored as occurring some, a lot, most or all the time over the past 4 weeks)	936	15.87
suicidal thoughts occurring some, a lot, most or all the time over the past two weeks	613	10.39
suicide plans occurring some, a lot, most or all the time over the past two weeks	247	4.19
suicide attempts over the past two weeks	133	2.26
current suicidal thoughts and/or behaviors (endorse any wishes to be dead, thoughts, plans or attempts over the past two to four weeks)	1071	18.16
lifetime suicide attempts	343	5.82
designated tier 1 (high risk) *	3015	51.11
Current mental health treatment		
medication for depression, anxiety or stress	1176	19.94
mental health counseling or psychotherapy	801	13.58
medications and/or therapy	1580	26.79

* Tier 1: Current suicidal thoughts and behaviors; a PHQ-9 score of 15 or higher with feelings of intense anxiety, anger, hopelessness, desperation or feeling out of control “a lot of the time” or “most of the time” or feelings of nervousness, annoyance, stress, loneliness or having arguments “most of the time”; a PHQ-9 score of 10–14 with a history of prior suicide attempts, problems related to alcohol or drug use, disordered eating behaviors or indications that current problems were making it somewhat difficult to function.

**Table 2:** Suicidal thoughts, behaviors and tiers by demographics, role and depression severity.

	Thoughts *N* = 613		Plans *N* = 247		Actions *N* = 133		PHQ-9 Item 9 *N* = 936		Current Suicidal Thoughts and/or Behaviors * N = 1071		Lifetime Attempts *N* = 343		Rated Tier 1 ** N = 3015	
	*N*	%	*N*	%	*N*	%	*N*	%	*N*	%	*N*	%	*N*	%
Age														
age < 40 years	366	10.54	129	3.72	87	2.51	569	16.39	638	18.39	212	6.11	1819	52.41
age ≥ 40 years	178	9.59	89	4.80	30	1.62	257	13.85	306	16.49	102	5.50	888	47.84
Gender														
female	398	9.55	155	3.72	94	2.25	636	15.3	723	17.34	254	6.14	2215	53.13
male	188	12.1	78	5.02	33	2.12	259	16.7	303	19.49	72	4.66	691	44.44
Race														
Black	17	11.64	8	5.48	6	4.11	21	14.38	29	19.86	16	10.96	79	54.11
White	280	9.39	102	3.42	52	1.74	412	13.9	482	16.17	135	4.56	1395	46.80
Hispanic	74	11.5	32	4.99	15	2.34	116	18.1	127	19.81	48	7.58	372	58.03
Asian or Pacific Islander	116	11.0	49	4.64	30	2.84	200	19	219	20.76	77	7.35	589	55.83
other	126	11.72	56	5.21	30	2.79	187	17.4	214	19.91	67	6.23	580	53.95
Role														
medical student	66	9.72	23	3.39	16	2.36	81	12	96	14.14	16	2.36	264	38.88
pharmacy student	26	8.39	15	4.84	10	3.23	52	16.8	57	18.39	16	5.18	146	47.10
house staff	83	9.62	16	1.85	14	1.62	121	14	138	15.99	33	3.82	421	48.78
nursing	142	10.8	60	4.57	31	2.36	231	17.7	257	19.56	114	8.76	766	58.38
physician	31	8.38	8	2.16	4	1.08	42	11.4	51	13.78	2	5.41	150	40.54
other	256	11.2	120	5.25	54	2.36	394	17.3	454	19.85	155	6.87	1224	53.53
Depression severity														
PHQ-8 < 10	118	3.27	46	1.27	33	0.91	182	5.04	251	6.95	102	2.82	995	27.53
PHQ-8 ≥ 10	495	21.67	201	8.8	100	4.38	754	33.01	820	35.9	241	10.55	2020	88.44

* Thoughts, plans, actions and/or PHQ item 9. ** Current suicidal thoughts and behaviors; a PHQ-9 score of 15 or higher with feelings of intense anxiety, anger, hopelessness, desperation or feeling out of control “a lot of the time” or “most of the time” or feelings of nervousness, annoyance, stress, loneliness or having arguments “most of the time”; a PHQ-9 score of 10–14 with a history of prior suicide attempts, problems related to alcohol or drug use, disordered eating behaviors or indications that current problems were making it somewhat difficult to function.

**Table 3:** Current mental health treatment by demographics, role, and depression severity.

	Current Medication for Depression, Anxiety and/or Stress		Current Mental Health Counseling or Psychotherapy		Current Medications and/or Therapy	
	*N*	%	*N*	%	*N*	%
Demographics						
% age < 40 years	608	17.52	463	13.34	857	24.70
% age ≥ 40 years	463	24.95	272	14.66	585	31.52
% female	903	21.66	592	14.2	1198	28.74
% male	233	14.98	179	11.51	329	21.16
% Black/African American	30	20.55	18	12.33	37	25.34
% White	712	23.88	438	14.69	926	31.06
% Hispanic	154	24.02	106	16.54	202	31.51
% Asian or Pacific Islander	112	10.62	115	10.9	190	18.01
% other	168	15.63	124	11.53	225	20.93
Role						
% medical student	75	11.05	98	14.43	137	20.18
% pharmacy student	34	10.97	35	11.29	53	17.1
% house staff	142	16.45	61	7.07	173	20.05
% nursing	348	26.52	211	9.23	451	34.38
% physician	64	17.3	37	10	85	22.97
% other	490	21.43	352	15.39	657	28.73
Depression Severity						
$ PHQ-8 < 10	512	14.17	419	11.59	761	21.06
% PHQ-8 ≥ 10	664	29.07	382	16.73	819	35.86

**Table 4:** Associations between clinical predictors and current suicidal thoughts and behaviors.

Predictor	Odds Ratio	95% Confidence Interval	Standard Error	z-Score
Model: depression				
PHQ-8 score	1.23 *	1.21, 1.25	0.01	25.83
Lifetime suicide attempt	3.17 *	2.41, 4.18	0.45	8.23
Model: maladaptive coping				
Drinking more than usual	2.07 *	1.62, 2.64	0.26	5.81
Other substance use	2.61 *	1.57, 4.34	0.68	3.69
Eating out of control	2.21 *	1.86, 2.62	0.19	9.02
Model: intense affective states				
Nervous	1.36 *	1.08, 1.71	0.16	2.66
Annoyed	1.16	0.95, 1.42	0.12	1.44
Stress	1.87 *	1.49, 2.35	0.22	5.41
Fighting	1.30	1.03, 1.62	0.15	2.25
Anxious	1.09	0.88, 1.33	0.11	0.78
Lonely	2.48 *	2.04, 3.01	0.25	9.17
Angry	0.99	0.78, 1.26	0.12	−0.07
Hopeless	3.04 *	2.41, 3.83	0.36	9.45
Desperate	1.38	1.07, 1.78	0.18	2.52
Out of control	1.07	0.86, 1.33	0.12	0.63

Note: * indicates *p* < 0.0125. All models included demographic covariates (age, gender, race/ethnicity and position). Lifetime suicide attempts were coded as 0 = no attempts, 1 = one or more attempts. Predictors in the maladaptive coping model were all coded as 0 (not at all; some of the time) or 1 (a lot of the time; most or all the time). Predictors in the intense affective states model were treated as continuous variables.

**Table 5:** Associations between clinical predictors and current mental health treatment status.

Predictor	Odds Ratio	95% Confidence Interval	Standard Error	z-Score
Model: depression				
PHQ-8 score	1.08 *	1.06, 1.09	0.01	11.36
Lifetime suicide attempt	1.76 *	1.32, 2.35	0.26	3.84
Current suicidality	1.14	0.94, 1.38	0.11	1.29
Model: maladaptive coping				
Drinking more than usual	1.50 *	1.19, 1.89	0.18	3.40
Other substance use	1.80	1.08, 3.00	0.47	2.25
Eating out of control	1.86 *	1.59, 2.18	0.15	7.66
Model: intense affective states				
Nervous	1.25 *	1.05, 1.48	0.11	2.53
Annoyed	1.14	0.97, 1.33	0.09	1.58
Stress	1.30 *	1.10, 1.54	0.11	3.05
Fighting	0.85	0.69, 1.04	0.09	−1.57
Anxious	1.59 *	1.34, 1.90	0.14	5.20
Lonely	1.23	1.03, 1.47	0.11	2.30
Angry	0.80	0.64, 1.00	0.09	−1.99
Hopeless	0.86	0.69, 1.08	0.10	−1.29
Desperate	1.36	1.06, 1.74	0.17	2.46
Out of control	1.16	0.96, 1.42	0.12	1.51

Note: * indicates *p* < 0.0125. All models included demographic covariates (age, gender, race/ethnicity, and position). Lifetime suicide attempt was coded as 0 = no attempts, 1 = one or more attempts; current suicidality was coded as 0 = no, 1 = yes. Predictors in the maladaptive coping model were all coded as 0 (not at all; some of the time) or 1 (a lot of the time; most or all of the time). Predictors in the intense affective states model were treated as continuous variables.

## Data Availability

The raw data supporting the conclusions of this article belong with the American Foundation for Suicide Prevention.

## Supplementary Materials

The following supporting information can be downloaded at: https://www.mdpi.com/article/10.3390/jcm14020574/s1, Table S1: Association between demographic factors and current suicidality; Table S2: Association between demographic factors and receiving mental health treatment.
