# Supplementary material for: Suicidal Thoughts and Behaviors Among Health Care Trainees, Staff and Faculty at an Academic Medical Center
Source: J Clin Med. 2025 Jan 17;14(2):574. doi: 10.3390/jcm14020574 (PMC11765537; doi:10.3390/jcm14020574)
Supplement: Supplementary file 1 [file jcm-14-00574-s001.zip › jcm-3368509-supplementary.pdf]

Supplementary Table S1. Association between demographic factors and current suicidality.

Predictor	Odds Ratio	95% confidence interval	Standard error	z-score
**Age**	**0.98**	**0.97, 0.99**	**0.01**	**−4.39**
**Female gender**	**0.80**	**0.68, 0.94**	**0.07**	**−2.64**
Race/ethnicity				
Black	1.46	0.95, 2.24	0.32	1.74
**Asian**	**1.38**	**1.14, 1.68**	**0.14**	**3.24**
Hispanic/Latino	1.17	0.94, 1.45	0.13	1.43
**Other background**	**1.45**	**1.15, 1.82**	**0.17**	**3.18**
Role				
Medical student	0.69	0.44, 1.08	0.16	−1.63
Pharmacy student	0.94	0.58, 1.53	0.23	−0.25
House staff	0.88	0.58, 1.33	0.18	−0.61
Nursing staff	1.52	1.05, 2.22	0.29	2.19
Other staff	1.45	1.01, 2.09	0.27	2.00

Bolded rows indicate $p < .0125$. Gender was coded as 0 = male, 1 = female. Non-Hispanic White was the reference group for race/ethnicity and physician was the reference group for role.

Supplementary Table S2. Association between demographic factors and receiving mental health treatment. .

Predictor	Odds Ratio	95% confidence interval	Standard error	z-score
Age	1.01	1.00, 1.01	0.01	1.65
**Female gender**	**1.42**	**1.22, 1.66**	**0.11**	**4.55**
Race/ethnicity				
Black	0.80	0.54, 1.19	0.16	−1.11
**Asian**	**0.53**	**0.44, 0.64**	**0.05**	**−6.57**
Hispanic/Latino	0.86	0.71, 1.03	0.08	−1.61
Other background	0.77	0.62, 0.94	0.08	−2.50
Role				
Medical student	0.84	0.58, 1.20	0.15	−0.96
Pharmacy student	0.77	0.50, 1.19	0.17	−1.18
House staff	0.85	0.61, 1.19	0.15	−0.95
**Nursing staff**	**1.58**	**1.18, 2.14**	**0.24**	**3.02**
Other staff	1.17	0.88, 1.55	0.17	1.06

Bolded rows indicate $p < .0125$. Gender was coded as 0 = male, 1 = female. Non-Hispanic White was the reference group for race/ethnicity, and physician the reference group for role.
